# *ARF1* ‐Related Diseases in China: The Initial Study of Phenotype and Molecular Profile

**DOI:** 10.1111/jcmm.70655

**Published:** 2025-07-21

**Authors:** Ruofei Lian, Gongao Wu, Liang Jin, Shichao Zhao, Ling Gan, Lijun Wang, Mengchun Li, Ruirui Liang, Tianming Jia, Yan Dong

**Affiliations:** ^1^ Department of Pediatrics The Third Affiliated Hospital of Zhengzhou University Zhengzhou China; ^2^ Henan Key Laboratory of Child Brain Injury and Henan Pediatric Clinical Research Center The Third Affiliated Hospital and Institute of Neuroscience Zhengzhou China; ^3^ NHC Key Laboratory of Birth Defects Prevention Zhengzhou China

**Keywords:** ADP‐ribosylation factor 1, drug‐refractory epilepsy, exome sequencing, gene function analysis

## Abstract

Background: The ADP‐ribosylation factor 1 (*ARF1*) gene encodes a protein which plays a critical role in intra‐Golgi transport. Clinical evidence suggests that individuals harbouring variants in the *ARF1* gene display a consistent set of phenotypic features, including intellectual disability, microcephaly, epilepsy, and periventricular nodular heterotopia (PVNH). Methods: This study describes the case of a 6‐year and 5‐month‐old female presenting with focal seizures on a fixed side that were resistant to various anti‐seizure medications. The genetic aetiology was elucidated through exome sequencing of the pedigree. The pathogenicity of the identified variant was subsequently assessed using molecular dynamics structural analysis, western blotting, and co‐immunoprecipitation techniques. Results: A *de novo* variant, c.509T > C (p.Leu170Pro), was detected in the *ARF1* gene, and functional analysis demonstrated that this modification is anticipated to hinder its association with the Golgi‐localising, γ‐adaptin ear homology domain and ARF‐binding protein, thereby playing a role in the pathogenesis of the disease. Conclusion: This study introduces the initial instance of *ARF1*‐related disease in China, wherein the patient is without the presence of PVNH. The findings add novel clinical phenotypes to the range of *ARF1*‐related diseases, and an investigation into the potential pathogenic mechanisms of this condition was conducted by confirming the deleterious impacts of the variant.

## 1 Introduction

In 2016, Ge et al. [1] identified 3 *de novo* missense variants within the GDP/GTP‐binding domain of the *ARF1* gene in three unrelated patients with PVNH, highlighting similar clinical characteristics among them. Subsequently, de Sainte Agathe et al. [2] reported 17 patients with *ARF1* variants confirming the role of *ARF1* in an autosomal‐dominant syndrome characterised by severe intellectual disability (ID), microcephaly, seizures and PVNH. This syndrome results from the haploinsufficiency of *ARF1* that impaired neuronal migration [3]. The *ARF1* gene belongs to the human *ARF* gene family, which comprises small guanine nucleotide‐binding proteins involved in various membrane trafficking events [4]. Based on amino acid sequence homology, this family includes five ARF proteins (ARF1 and ARF3–ARF6) [5], grouped into three classes: Class I (ARF1 and ARF3, sharing 96% identity), Class II (ARF4 and ARF5, 90% identity to each other and 82% to ARF1) and Class III (ARF6, 68% identity to ARF1) [6]. Both Class I and II ARFs primarily localise to the Golgi apparatus and play pivotal roles in Golgi transport in vivo [6, 7]. Among these, ARF1 is the most abundant and extensively studied. Notably, ARF1 and ARF3 interact specifically with the GTP‐bound form and associate with a novel family of potential ARF effectors known as GGA1–GGA3 (Golgi‐localising, gamma‐adaptin ear homology domain, ARF‐binding protein). These effectors are predominantly localised to the trans‐Golgi network (TGN) or related compartments [4]. To date, *ARF1*‐related neurological disorders have been sparsely documented, with only 22 cases reported worldwide. By presenting the first case in China with drug‐refractory epilepsy (DRE) but without PVNH, we aim to enrich the phenotypic spectrum of *ARF1*‐related diseases and enhance our understanding of underlying pathogenic mechanisms.

## 2 Materials and Methods

### 2.1 Participant Consent and Ethical Approval

This study involved a patient from a Han family who was diagnosed with *ARF1*‐related diseases through clinical evaluations and genetic testing. The patient received treatment in the inpatient department of the Third Affiliated Hospital of Zhengzhou University. Written informed consent was obtained from the family members for participation in this study. The study followed the CARE guidelines and was conducted in accordance with the Declaration of Helsinki [8]. Approval was also obtained from the Ethics Committee of The Third Affiliated Hospital of Zhengzhou University (2021‐062‐01).

### 2.2 Exome Sequencing

For genomic DNA extraction, 2 mL of peripheral blood (containing the anticoagulant EDTA) was collected from the patient, her brother and her parents. The extraction kit from Kangwei Century (Shanghai, China) was utilised as per the manufacturer's instructions. Following quality control and library preparation, DNA fragments within the target region were enriched, and a whole‐exome library was constructed. Probes were captured using the xGen Exome Research Panel v1.0 (IDT Company, USA). Sequencing was performed on an Illumina NovaSeq 6000 series sequencer (Illumina, San Diego, CA, USA). Post‐sequencing, the data were aligned with the human reference genome (GRCh37/hg19) using the Burrows‐Wheeler Aligner software (version 0.59). The Genome Analysis ToolKit (version 4.0.4.0) was used to filter and identify SNPs and indels, ensuring the detection of high‐quality, reliable variants. These variants were classified following the American College of Medical Genetics (ACMG) guidelines [9]. MEGA7.0 software was used to analyse the conservation of amino acid variation sites and PyMOL to model protein structure and analyse the effect of the variation on spatial protein structure.

### 2.3 Experimental Steps of Functional Assay

#### 2.3.1 Molecular Dynamics Simulation Structure

The protein structures of ARF1 and GGA3 were obtained from the RCSB PDB database (https://pdbus.org/, PDB: 8SDW and 1P4U, respectively). The variant ARF1 Alphafold protein structure was used for building (https://alphafold.ebi.ac.uk/). The ZDOCK algorithm was used for molecular docking, and Gromacs5.14 for MD simulation (http://www.gromacs.org/). Data result graphs were generated by using Pymol 2.5 software (https://pymol.org/2/) and Origin8.5 software. The root mean square deviation of the complex protein was calculated using the gmx rms tool, and the structure map was drawn using PyMOL. The free binding energy between proteins was calculated using the MM‐PBSA algorithm.

#### 2.3.2 Plasmid Construction and Cell Transfection

Human 293 T cells, sourced from the Shanghai cell bank, were cultured in high‐glucose DMEM (Gibco, USA) supplemented with 10% foetal bovine serum. These cells were incubated in an environment of 5% CO_2_ at 37°C. Then plasmid transfection occurred when cell density reached 60% confluence in a six‐well plate by using Lipofectamine 2000 (Thermo Fisher Scientific, United States). The wild type *ARF1* (*ARF1*‐WT) was constructed using the pECMV‐3 × FLAG‐N vector following the protocol provided with the Phanta Max Super‐Fidelity DNA Polymerase (Vazyme, #P505) kit. The amplification primers used were: Forward: 5′‐aaggatgacgatgacaagcttATGGGGAACATCTTCGCCA‐3′ and Reverse: 5′‐tgctggatatctgcagaattcTCACTTCTGGTTCCGGAGCTG‐3′. Subsequently, the variant *ARF1* (*ARF1*‐MUT) group was generated using the Mut Express MultiS Fast Mutagenesis Kit V2 (Vazyme, #C215), with the following primers: Forward: 5′‐aaggatgacgatgacaagcttATGGGGAACATCTTCGCCA‐3′; Reverse: 5′‐TGCTGGATATCTGCAGAATTCTCACTTCTGGTTCCGGAGCTGATTGGACAGCCAGTCCGGTCCTTCATA‐3′. Post‐sequencing, plasmid amplification was carried out.

#### 2.3.3 Western Blot (WB)

The transfected *ARF1*‐WT and *ARF1*‐MUT cells were lysed using protein lysate (#P0013, Beyotime, China), and we analysed protein expression using a WB assay. After lysis, the cell lysates were subjected to SDS‐PAGE electrophoresis, transferred onto PVDF membranes, and blocked with TBS containing 5% skim milk powder and 0.05% Tween‐20 (Beyotime, China) for 2 h. The membranes were then probed overnight at 4°C with primary antibodies: DYKDDDDK Tag (9a3) Mouse mAb (Cat # 8146, dilution 1:1000, Cell Signaling Technology, USA) and β‐Actin (D4C6R) Mouse mAb (Cat # 3700, dilution 1:1000, Cell Signaling Technology, USA). This was followed by incubation with the secondary antibody, anti‐mouse IgG, HRP‐linked (Cat # 7076, dilution 1:5000, Cell Signalling Technology, USA). Detection was subsequently performed. Band analysis was conducted using Image J v1.46 software (NIH, Bethesda, USA), and statistical analysis and graphing were done using GraphPad Prism 8 (GraphPad Software, San Diego, USA). Statistically significant differences were noted at *p* < 0.0001, and the experiment was repeated three times.

#### 2.3.4 Co‐Immunoprecipitation (Co‐IP)

A Co‐IP experiment was conducted to examine the interaction between the modified ARF1 protein and GGA3 protein. Flag‐tagged *ARF1* plasmids (both *ARF1*‐WT and *ARF1*‐MUT) and Myc‐tagged GGA3 were transfected separately into 293 T cells and co‐transfected for 48 h. After cell lysis, 400 μl of the supernatant was reserved. Of this, 100 μl was used as the Input sample, and the remaining 300 μl was processed using anti‐Flag immunomagnetic beads (B26101, Selleck, Germany) following the manufacturer's protocol for bead washing, sample binding and subsequent washing steps. Finally, 50 μL of 1× protein loading buffer was added, and the samples were boiled for 5 min to elute the proteins. The resulting supernatant served as the IP samples, and all samples were analysed by SDS‐PAGE.

## 3 Results

### 3.1 Clinical Findings

**FIGURE 1:**
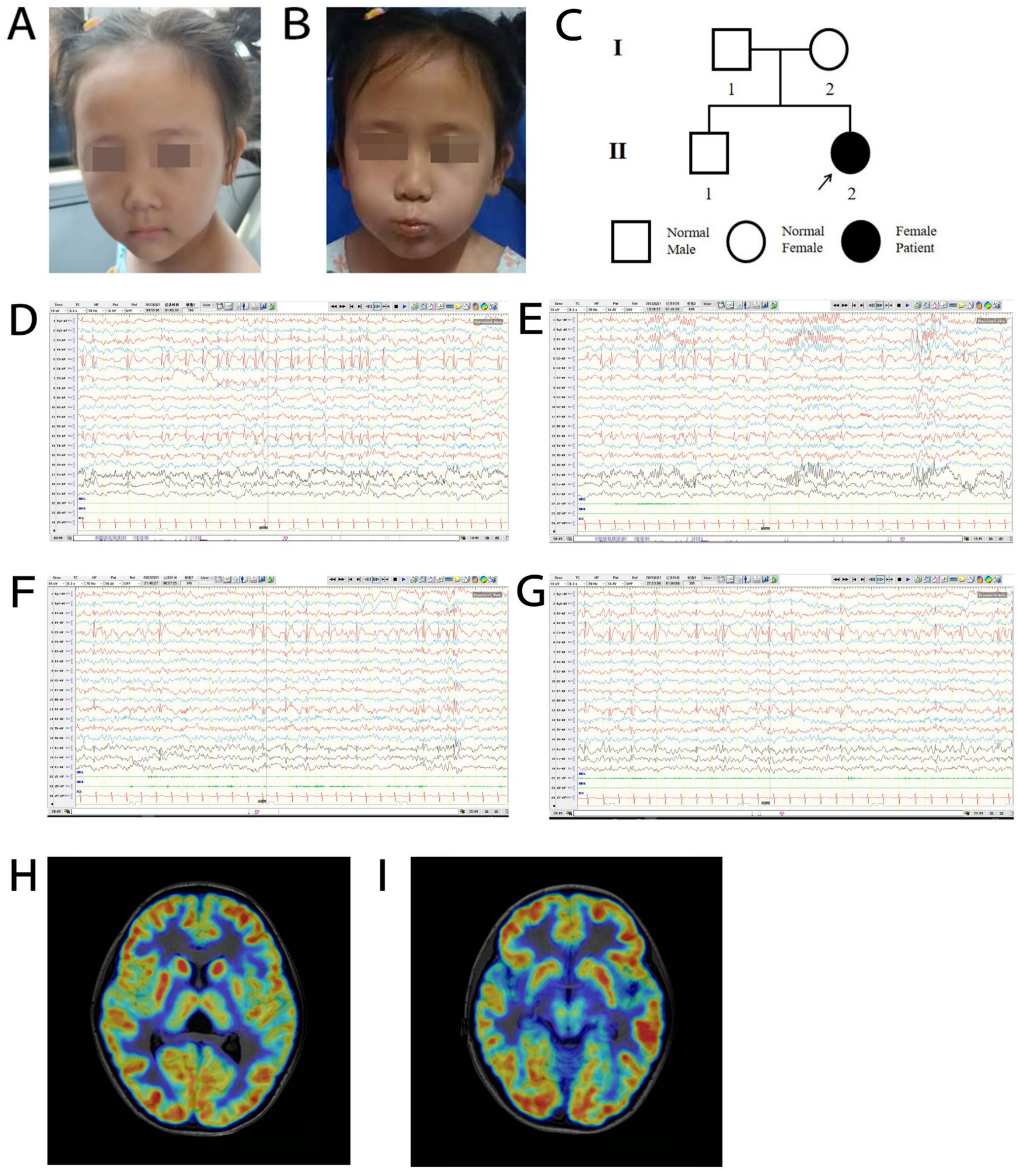
Phenotype of our patient (A and B). Genealogy map of the patient's family (C). VEEG from February 21, 2022, when the patient was 4 years old (D–G, abnormal discharge in the left central, parietal and temporal regions of the brain). MRI and PCT‐CT fusion images of the patient (H and I). MRI, magnetic resonance imaging; PCT‐CT, Positron emission tomography–computed Tomograph VEEG, video electroencephalograph..

The patient, a female aged 6 years and 5 months, was born via caesarean section at term to non‐consanguineous parents with no apparent personal or family medical history (Figure 1A–C). Prior to the onset of symptoms, her growth and developmental milestones were within normal limits, including head control at 3 months, independent sitting at 6 months and walking at 1 year of age. In December 2020, at 3 years of age, the patient experienced a seizure characterised by right limb tremors, loss of consciousness, and foaming at the mouth following 3 days of diarrhoea. The seizure lasted approximately 2 min. Subsequent episodes involved brief tremors of the patient's right fingers without loss of consciousness, lasting from a few seconds to several minutes. The frequency of these epileptic seizures was approximately twice per month. The patient was diagnosed with epilepsy in December 2021 and was prescribed a regimen of oral medications including oxcarbazepine (OXC), levetiracetam (LEV), valproate (VPA) and perampanel (PER) sequentially. However, these medications proved to be ineffective in controlling the seizures. Ultimately, successful seizure management was achieved with a combination of therapy of VPA (18.6 mg/kg/day) and CZP (0.03 mg/kg/day). At the time of physical examination in April 2024, the child was 115 cm tall (−1 SD to 0 SD), weighed 29 kg (+2 SD to +3 SD) and did not have special facial features. The Griffiths Neurodevelopmental Assessment indicated that the individual exhibited adequate language, motor and logical thinking skills but showed deficiencies in social skills. Furthermore, her responsiveness to simple commands was below average, and there was a significant delay in hand‐eye coordination. Limited dexterity was observed in both hands, particularly the right hand. Multiple Video‐Electroencephalographies (VEEG) revealed abnormal discharges in the central, parietal and temporal lobes of the left hemisphere (Figure 1D–G). However, magnetic resonance imaging (MRI) and enhanced scanning techniques were conducted, and further diagnostic imaging, such as enhanced scans, brain function imaging and Positron Emission Tomography‐Computed Tomography (PET‐CT), revealed only mild atrophy in the left hippocampus and the inner aspect of the adjacent temporal lobe, with no significant abnormalities or evidence of PVNH.

### 3.2 Molecular Analysis

Genetic analysis utilising pedigree‐base exome sequencing revealed the presence of a *de novo* missense variant c.509T > C (p.Leu170Pro) in the *ARF1* gene (NM_001658.4), which was absent in other members of the familial cohort. Several variant prediction tools indicate a potential deleterious effect of this variant, and it demonstrates a REVEL score of 0.899 (PP3_Moderate) (Table 1) [10]. Furthermore, the variant is *de novo* and the associated disease of the gene corresponds with the patient's clinical manifestations, albeit with moderate specificity (PS2_Moderate). The Genome Aggregation Database (gnomAD v.4.1.0, http://www.gnomad‐sg.org/) reports this variant as unreported (PM2_Supporting) (Table 2). Furthermore, by establishing cell models for functional verification, the results indicated that this variation might lead to impairment of gene function (PS3_Supporting) [11]. The 2019 guidelines established by the American College of Medical Genetics and Genomics [9] categorised this newly identified variant has been upgraded to Likely Pathogenic with the potential to elucidate the patient's phenotype (PS2_Moderate, PM2_Supporting, PP3_Moderate, PS3_Supporting).

**TABLE 1:** *ARF1* c.509 T > C (p.Leu170Pro): Software prediction results.

Software	SIFT	Polyphen2_HDIV	Polyphen2_HVAR	Mutation taster	Mutation assessor	REVEL	LRT	CADD_raw
ARF1 c.509 T > C(p.Leu170Pro)	Deleterious	Deleterious	Likely deleterious	Disease‐causing	High probability of pathogenicity	0.899	Deleterious	5.327

**TABLE 2:** *ARF1* c.509 T > C (p.Leu170Pro): Database inclusion in normal population.

Database	gnomAD_ exome_ALL	gnomAD_ exome_East Asia	gnomAD_ genome_ALL	gnomAD_ genome_East Asia	ExAC_ALL	ExAC_East Asia
*ARF1* c.509 T > C(p.Leu170Pro)	—	—	—	—	—	—

*Note:* Absent or extremely rare in population databases.

### 3.3 Functional Assay

#### 3.3.1 Structural Model

**FIGURE 2:**
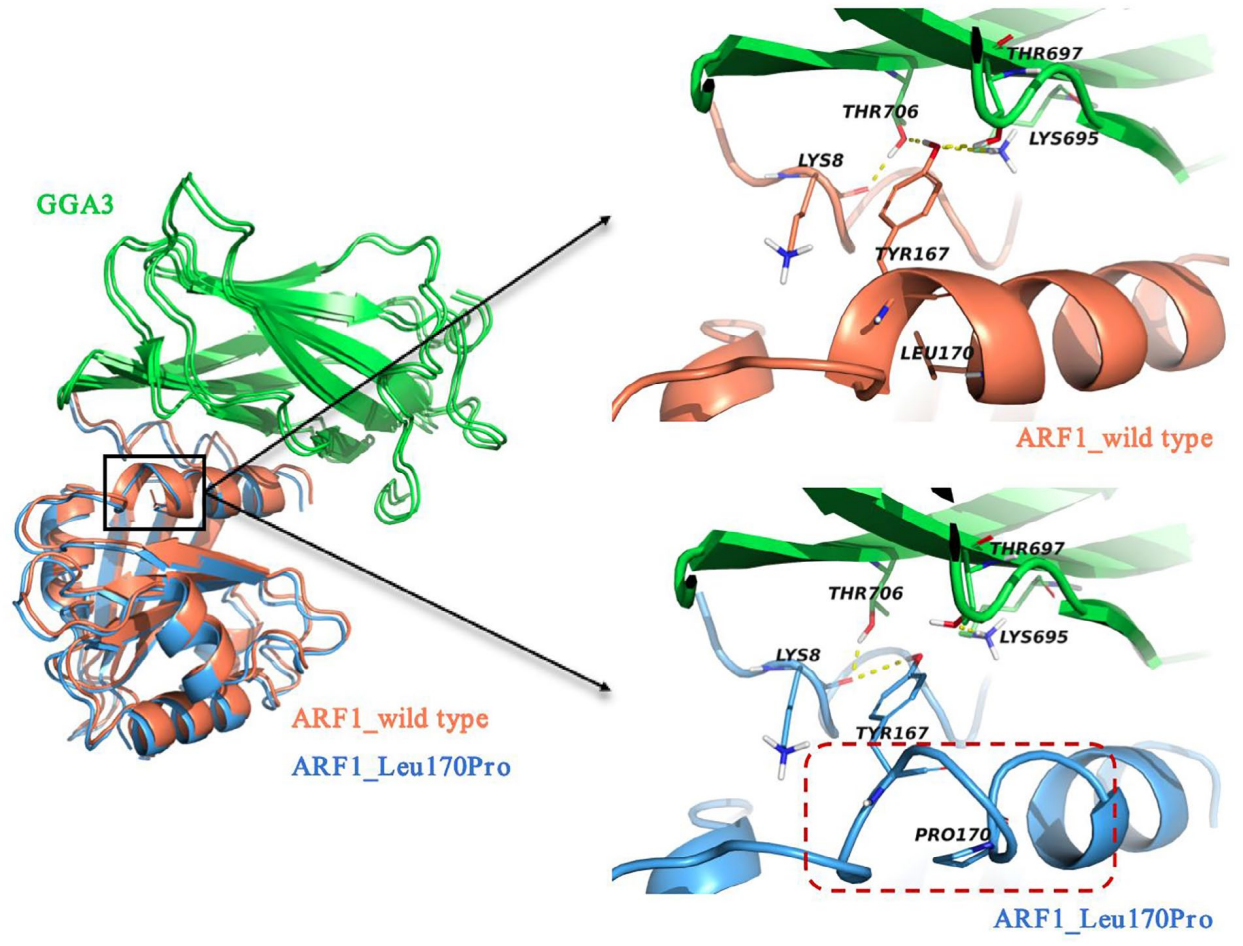
Molecular dynamics simulation of the complex structure of ARF1 and GGA3 proteins showed that the Leu170Pro variant in ARF1 resulted in the change of the α‐helix structure to the Loop structure (shown in the red box), and the variant also destroyed the hydrogen bonding between the adjacent Tyr167 residue and the GGA3 protein Thr706, Thr697 and Lys695 residues.

The ARF1–GGA3 complex structure was assembled and subsequently subjected to molecular dynamics (MD) simulation. The root mean square deviation (RMSD) values for both the wild‐type and variant structures converged to a common value of 1.70 Å. Examination of the protein's spatial configuration revealed that in the wild‐type ARF1, Leu170 is situated within an alpha‐helical motif at the C‐terminal region and does not directly interact with GGA3. Conversely, the adjacent residue Tyr167 can engage in hydrogen bonding interactions with GGA3 residues Thr706, Thr697 and Lys695. In the variant structure, the Leu170Pro variant leads to conformational changes shifting the secondary structure from an alpha‐helix to a loop. This alteration results in the formation of intramolecular hydrogen bonds between Tyr167 and Lys8, while disrupting existing hydrogen bonds with GGA3 at Thr706, Thr697 and Lys695, as detailed (Figure 2 and Table 3). The calculated free binding energy between wild‐type ARF1 and GGA3 was determined to be −91.97 kcal/mol, whereas this value increased to −68.82 kcal/mol for the variant ARF1‐GGA3 complex. This observation suggests that the variant may result in a diminished interaction with GGA3.

**TABLE 3:** Binding free energy analysis of ARF1‐GGA3 protein interaction.

Associative free energy contribution	Energy value of ARF1‐GGA3(kJ/moL)	Energy value of ARF1 (L170P)‐GGA3(kJ/moL)
△Evdw	−127.86	−92.27
△Eelec	−16.79	−22.53
△GGB	79.10	70.56
△GSurf	−26.43	−24.58
△Gbind	−91.97	−68.82

Abbreviations: △Evdw, the van der Waals effect; △Eelec, electrostatic interaction; △GGB, polar solvation energy; △GSurf, non‐polar solvation energy; △Gbind, total binding free energy of complex.

#### 3.3.2 Western Blot

**FIGURE 3:**
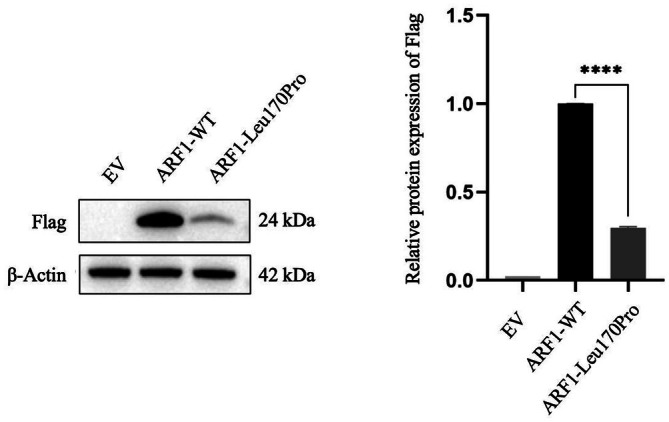
Western blot results showed that the expression of Flag‐tagged ARF1‐MUT group was a little low, which was significantly lower than that of Flag‐tagged ARF1‐WT group, about 70% (****p < 0.0001).

The findings indicated a significant increase in the expression of Flag‐tagged *ARF1*‐WT in the empty vector (EV) group compared to the *ARF1*‐MUT group. Moreover, histone expression was notably reduced in the *ARF1*‐MUT group in comparison to the *ARF1*‐WT group (Figure 3).

#### 3.3.3 Co‐Immunoprecipitation

**FIGURE 4:**
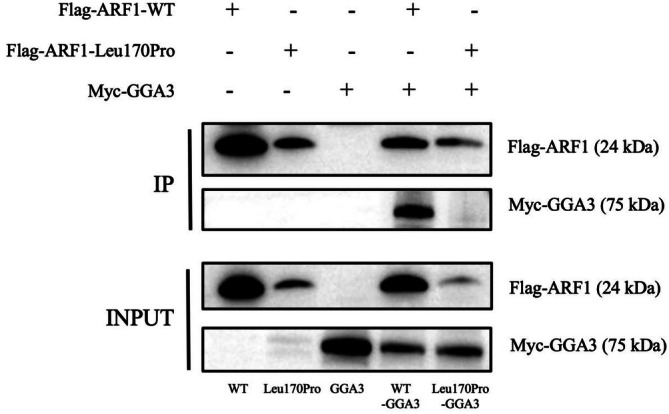
Wild type and mutant ARF1 were co‐transfected into 293 T cells with GGA3, and Flag protein was immunoprecipitated. The interaction between ARF1 and GGA3 was detected by western blotting, and results suggest that ARF1 variation may affect the interaction with GGA3.

The results obtained from the Input group demonstrate the presence of imprinting in all groups, thus confirming the typical expression of protein samples. In the IP group, the presence of Flag‐*ARF1*, Flag‐*ARF1*‐Leu170Pro and co‐transfected samples with Myc‐GGA3 was observed, consistent with the findings in the Input group. This suggests successful precipitation of the Flag‐tagged protein complexes. Expression of the Myc tag was identified in the co‐transfected complexes of Flag‐*ARF1* and Myc‐GGA3. However, a notable decrease in expression was noted in the complex of Flag‐*ARF1*‐Leu170Pro and Myc‐GGA3, indicating a potential impact of the *ARF1* variant (Figure 4).

## 4 Discussion

ADP‐ribosylation factor (ARF) functions as a key regulator of intracellular transport, overseeing the coordination of intracellular transport processes throughout different stages of the secretory pathway, from the endoplasmic reticulum‐Golgi interface to the plasma membrane. The activation of ARF is controlled by the alternating cycles of GTP/GDP exchange and GTP hydrolysis [12]. ARFs are classified into three subgroups based on their sequence similarity, with Type I comprising ARF1 and ARF3 [13]. ARF1 and ARF3 exhibit structural similarity with the exception of seven amino acids located at the N‐ and C‐terminal regions, implying a potential overlap in their functions and roles. Furthermore, both genes have been implicated in neurodevelopmental disorders, underscoring their potential pathogenic significance [12, 14]. This study presents the initial case of *ARF1*‐related disease in China, featuring drug‐refractory and focal epilepsy as well as mild growth delay in comparison to peers. Extensive imaging studies did not detect any indications of PVNH. The clinical manifestation observed in this case deviates from the previously documented range of *ARF1*‐related disorders.

Three novel missense variants in the *ARF1* gene, specifically c.103T > C (p.Tyr35His), c.379 A > G (p.Lys127Glu), and c.296G > A (p.Lys127Gly), were initially documented by Ge et al. in 2016 [1]. These variants were found to be linked to ID and PVNH, with affected individuals displaying additional symptoms such as abnormal white matter signals and epilepsy. Subsequently, Gana et al. [3] reported a case involving a girl with ID and PVNH, as well as her father, who both carried the *ARF1* gene variant c.234G > A (p.Trp78Ter). Notably, both individuals exhibited ectodermal dysplasia, mirroring the findings of the three cases reported by Ge et al., thus reinforcing the observed associations. In a recent extensive cohort study, 12 novel variants of the *ARF1* gene were discovered in 17 unrelated individuals, consisting of 10 missense variants, 1 frameshift variant and 1 splice variant. The observed clinical manifestations linked to these variants included ID, short stature, microcephaly, epilepsy and PVNH [2]. Only 30% of the patients in this cohort study were found to exhibit PVNH, with imaging studies revealing additional abnormalities such as microcephaly, hypoplasia of the corpus callosum, and polymicrogyria. Analysis of these cases indicated that children with PVNH or other brain malformations may not necessarily present with seizures. However, those who did exhibit seizures demonstrated a variety of brain structural abnormalities, with diverse types of seizures experienced. In the case under consideration, the child experienced localised seizures affecting solely the right side. Initially, it was hypothesised that the child's DRE may have a structural aetiology. Unlike previous cases, thorough imaging studies failed to identify any pathological lesions. We speculate that the imaging abnormalities in these children may exhibit variations based on the types and locations of genetic variants. All individuals with available information presented with varying degrees of ID, ranging from mild to severe. Our findings indicate that the Griffith neurodevelopmental assessment results for this child revealed a mild developmental delay. This observation aligns with clinical data recorded in similar cases previously. Variants in *ARF3* have been associated with generalised developmental delays or ID, frequently presenting with brain and bone irregularities such as microcephaly. As of now, a total of seven pathogenic missense variants have been documented [15, 16].

*ARF1* has been the subject of extensive research and is recognised for its pivotal role in the regulation of capsid protein complex assembly at vesicle budding sites [4]. Similar to other GTPases, ARF1 undergoes a cycle of activation and inactivation through GTP binding. In its GDP‐bound state, ARF1 is predominantly localised in the cytoplasm, whereas in its active GTP‐binding state, ARF1 associates with membranes via its N‐myristoylated amphiphilic alpha helix. The activation of ARF1 involves the interaction of its switch 1 (amino acids 42–52) and switch 2 (amino acids 70–85) regions [6]. Guanine nucleotide exchange factors (GEFs) mediate the exchange of GDP for GTP on ARF1, whereas GTPase‐activating proteins (GAPs) facilitate the hydrolysis of GTP back to GDP [5]. The fungal metabolite brefeldin A (BFA) inhibits ARF1 function by stabilising the formation of complexes between ARF1‐GDP and specific ARF1‐GEFs [17]. Coat proteins are the most significant effectors of ARF1, regulating both forward and retrograde transport within the Golgi and trans‐Golgi network (TGN) [5]. ARF1 regulates various coat proteins, such as coat protein complex I (COPI), adapter protein (AP) complexes (AP‐1, AP‐3, AP‐4) and GGA proteins (GGA1–GGA3) [18], by recruiting them to the membrane to facilitate the formation of transport vesicles and the encapsulation of specific cargo. Each of these coat proteins plays a unique role in mediating specific transport processes [6].

The existing genetic and clinical data highlight the significant involvement of Type I ARFs in the development of the nervous system, neuronal migration, and the maintenance of ongoing functionality. Dysregulation of these processes is associated with the pathophysiological pathways of ID, PVNH and seizures. In a study conducted by Ishida et al. [6] in 2023, a 9‐year‐old patient with a variant in the *ARF1* gene, specifically c.296G > A (p.Arg99His), presented with developmental delays, hypotonia, ID and stereotyped movements. Neuroimaging revealed hypoplasia of the corpus callosum and subcortical white matter abnormalities in the patient. It is noteworthy that this patient did not exhibit seizures or PVNH, which are commonly seen in individuals with *ARF1* variants. In contrast, a case study conducted by Ge et al. [1] in 2016 reported the same *ARF1* gene variant in a patient who presented with seizures and PVNH. Functional analysis conducted by Ishida et al. demonstrated normal expression levels and confirmed the localisation of the mutated protein in the Golgi apparatus. Nevertheless, this variant resulted in enlargement of the Golgi apparatus and exhibited increased affinity for GGA3 compared to the unaltered ARF1 protein.

These results suggest that the pathogenic process may entail constitutive activation resulting in changes in the Golgi apparatus and endosomes, rather than consistent phenotypic presentations in all *ARF1*‐related disorders. To enhance comprehension, additional studies with larger sample sizes and thorough functional verification are imperative. In order to clarify the distinct neuronal roles of *ARF1* and the pathophysiological implications of its pathogenic variants, we investigated the biological consequences of a novel *ARF1* gene variant in this child. This study utilises hazard prediction algorithms in gene detection, molecular dynamics simulation analysis, WB and Co‐IP. The findings indicated a potential decrease in expression levels and altered interactions with the GGA3 protein caused by the identified variant. Furthermore, research suggested that specific ARF1 amino acids, including Arg19, Tyr35, Thr48, Lys127, Phe51 and Arg99, may impact GTP/GDP exchange and the switch 1 and switch 2 regions, potentially resulting in functional changes [2]. The Leu170Pro variant identified in this study, situated proximal to the C‐terminal region, has been shown to disrupt structural conformation and impact interactions with GGA3, as evidenced by molecular dynamic simulations and immunoprecipitation assays. Current investigations indicate the intricate nature of ARF1 protein functionality, with distinct variants potentially resulting in diverse functional alterations.

At present, a definitive treatment for individuals diagnosed with this condition remains elusive. Instead, personalised symptomatic treatments are offered, such as the administration of anti‐seizure medications to manage seizures, with dosages adjusted according to the patient's response. Additionally, rehabilitation training is advised to target motor delays. It is anticipated that forthcoming research endeavours will formulate a treatment regimen that is both extensive and enduring. Genetic testing is advised for children displaying symptoms such as ID, microcephaly, seizures and PVNH in order to validate diagnoses. Families affected by this condition should also contemplate prenatal diagnosis for the purpose of acquiring comprehensive information.

## 5 Conclusion

The present study reports the initial identification of an *ARF1* gene variant in a Chinese paediatric patient with DRE, characterised by the absence of associated imaging abnormalities, a deviation from previously reported cases. The detrimental impact of this variant was assessed through protein structure analysis, WB, and Co‐IP, indicating potential implications for reduced expression levels and modified interactions with the GGA3 protein. It is crucial to acknowledge that the exogenous gene introduction utilised in our study may not accurately replicate the natural intracellular environment, thereby potentially impacting the precision of our results. Moreover, further functional investigations are warranted to clarify any morphological alterations in the Golgi apparatus linked to this variant.

## Author Contributions

**Ruofei Lian:** formal analysis (lead), methodology (supporting), writing – original draft (lead), writing – review and editing (equal). **Gongao Wu:** data curation (equal), software (lead), writing – review and editing (equal). **Liang Jin:** data curation (equal), methodology (lead), writing – review and editing (equal). **Shichao Zhao:** investigation (equal), writing – original draft (supporting). **Ling Gan:** data curation (equal), methodology (supporting). **Lijun Wang:** investigation (equal). **Mengchun Li:** software (supporting), writing – review and editing (equal). **Ruirui Liang:** writing – review and editing (equal). **Tianming Jia:** project administration (lead), validation (lead). **Yan Dong:** conceptualization (lead), funding acquisition (lead), resources (lead).

## Ethics Statement

This study was performed in line with the principles of the Declaration of Helsinki. Approval was granted by the Ethics Committee of the Third Affiliated Hospital of Zhengzhou University (No. 2021–062‐01).

## Consent

The parents of the patient provided written informed consent. The authors affirm that human research participants provided informed consent for publication of the images in Figure 1.

## Conflicts of Interest

The authors declare no conflicts of interest.

## Data Availability

DATA AVAILABILITY STATEMENTThe dataset supporting the conclusions of this article is available in: https://www.ncbi.nlm.nih.gov/clinvar/, accession numbers: SCV005061487. According to national legislation/guidelines, specifically the Administrative Regulations of the People's Republic of China on Human Genetic Resources (https://www.gov.cn/zhengce/content/2019‐06/10/content_5398829.htm, http://english.www.gov.cn/policies/latest_releases/2019/06/10/content_281476708945462.htm), no further datasets presented in this article are readily available. Requests to access the datasets should be directed to the corresponding author.
